# Plasmonic Amyloid Tactoids

**DOI:** 10.1002/adma.202106155

**Published:** 2021-10-17

**Authors:** Ye Yuan, Hamed Almohammadi, Julie Probst, Raffaele Mezzenga

**Affiliations:** ^1^ Department of Health Sciences and Technology ETH Zürich Zürich 8092 Switzerland; ^2^ Department of Chemistry and Applied Biosciences ETH Zürich Zürich 8093 Switzerland; ^3^ Department of Materials ETH Zürich Zürich 8093 Switzerland

**Keywords:** amyloid fibrils, fluorescence, gold nanorods, liquid crystals, plasmonics, self‐assembly

## Abstract

Despite their link to neurodegenerative diseases, amyloids of natural and synthetic sources can also serve as building blocks for functional materials, while possessing intrinsic photonic properties. Here, it is demonstrated that orientationally ordered amyloid fibrils exhibit polarization‐dependent fluorescence, and can mechanically align rod‐shaped plasmonic nanoparticles codispersed with them. The coupling between the photonic fibrils in liquid crystalline phases and the plasmonic effect of the nanoparticles leads to selective activation of plasmonic extinctions as well as enhanced fluorescence from the hybrid material. These findings are consistent with numerical simulations of the near‐field plasmonic enhancement around the nanoparticles. The study provides an approach to synthesize the intrinsic photonic and mechanical properties of amyloid into functional hybrid materials, and may help improve the detection of amyloid deposits based on their enhanced intrinsic luminescence.

## Graphical abstract

Hybrid liquid crystalline tactoids consisting of gold nanorods aligned by amyloid fibrils exhibit enhanced fluorescence with polarization and wavelength sensitivity. This work demonstrates how anisotropic colloidal self‐assembly couples plasmonic nanoparticles with the intrinsic photonical properties of amyloids.

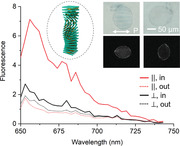

## 1 Introduction

Amyloid fibrils (AFs) are formed by β‐sheet rich polypeptides stacking up into filamentous structures.^[^ ^1^, ^2^, ^3^, ^4^ ^]^ The extensive network of hydrogen bonds within these supramolecular protein assemblies results in the chemical stability and mechanical rigidity of the fibrils,^[^ ^5^, ^6^ ^]^ which may contribute to the pathology of amyloid aggregation in neurodegenerative diseases.^[^ ^1^, ^2^ ^]^ However, this robustness is desirable in other biological functions,^[^ ^7^ ^]^ such as biofilm formation, cell adhesion, hormone release, etc. Aided by their proteinaceous nature, especially when their composition arises from nontoxic or even food‐grade proteins, amyloid fibrils have also gained popularity in fabricating various types of artificial functional materials for water purification, biosensing, photovoltaics, or even antiviral filtration.^[^ ^7^, ^8^, ^9^, ^10^ ^]^ The potential of amyloid fibrils in artificial materials can be further enriched by their ability to form anisotropic assemblies. Like many other rod‐like colloidal particles, aqueous suspensions of amyloid fibrils can self‐assemble into phases with long‐range orientational ordering, i.e., liquid crystals (LCs), driven by entropy.^[^ ^11^, ^12^, ^13^, ^14^ ^]^ In addition to the common nematic phase where there is no positional ordering, the inherent chirality of the fibrils also leads to cholesteric phases with helical twisting alignment of fibrils by controlling the fibrils length distribution and confinement.^[^ ^15^, ^16^ ^]^ These ordered states lead to anisotropy in the mechanical, rheological, and optical properties of the fibrils assemblies in meso‐ and macroscale, which, however, has yet to be taken full advantage of in the fabrication of functional materials.^[^ ^7^, ^8^ ^]^

An emerging research interest is the photonic properties of amyloid fibrils. First observed as UV‐induced blue‐green luminescence,^[^ ^17^ ^]^ it has been extended to UV–vis–NIR range^[^ ^18^ ^]^ and wavelength‐dependent nonlinear absorption is also discovered in AFs.^[^ ^19^ ^]^ Although the exact origin of the intrinsic fluorescence is still under debate, studies have demonstrated that it can be used to monitor the growth of the fibrils^[^ ^20^ ^]^ or to image amyloid deposits in a noninvasive and contrast‐agent‐free fashion.^[^ ^18^, ^21^ ^]^ These findings may contribute to the diagnosis and treatment of human amyloidosis, as well as the development of novel bionanomaterials. However, the intrinsic fluorescence suffers from low quantum efficiency and weak signal to noise ratio, and therefore, hinders its potentials in practical applications.^[^ ^18^, ^19^, ^20^, ^21^, ^22^ ^]^

In this work, we study the intrinsic fluorescence of amyloid fibrils in orientationally ordered phases and investigate their interaction with plasmonic nanoinclusions codispersed with the fibrils. We show that the fluorescence from the fibrils is dependent on the polarization of the excitation when they are in microdroplets with liquid crystalline ordering, i.e., tactoids. Then gold nanorods (GNRs) are introduced to the colloidal suspensions of AFs, forming hybrid tactoids with GNRs aligned by the LC orientation field of the fibrils. This alignment manifests through the selective activation of surface plasmon resonance (SPR) absorption bands of GNRs, resulting in color changes when illuminated with polarized white light. Furthermore, enhanced fluorescence from the hybrid material is observed due to the coupling between the GNRs’ plasmonic effect and the fibrils’ intrinsic fluorescence. The enhanced fluorescence is strongly dependent on the wavelength and polarization of the incident light, which is confirmed by numerical simulations of the electromagnetic field distribution in the vicinity of GNRs. We point out the unusual higher concentration of GNRs within the hybrid tactoids compared to that in the isotropic surrounding and will discuss how the polarization dependence is related to the origin of the intrinsic luminescence of amyloid fibrils. These results provide new directions for exploiting the ordered states and photonic properties of the fibrils to fabricate functional composite materials.

## 2 Results and Discussion

**Figure 1:**
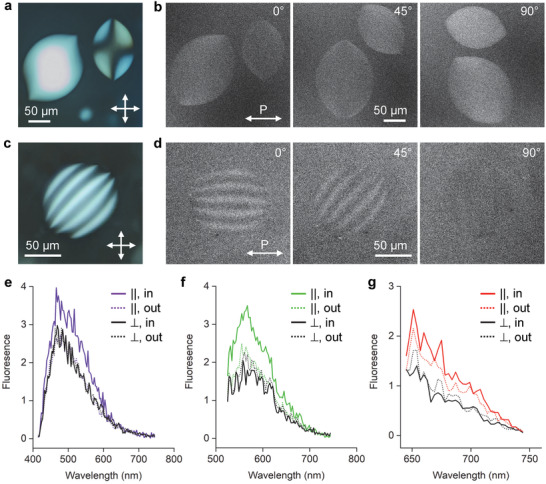
Polarization‐dependent fluorescence of amyloid fibrils tactoids. a) Polarizing microscopy image of bipolar tactoids. The white double arrows represent the crossed polarizers. b) Corresponding fluorescence images taken at different sample orientation with respect to the fixed polarization of the excitation light. c,d) Polarizing and fluorescence microscopy images of a cholesteric tactoid. All the fluorescence images are obtained with a 405 nm excitation laser and the linear laser polarization is marked with *P*. e–g) Polarization‐dependent fluorescence spectra measured within and outside of tactoids using 405 nm (e), 514 nm (f), and 633 nm (g) excitation light. Colored lines are spectra obtained with the polarization of excitation parallel to the fibril orientation and black lines are spectra obtained with the polarization of excitation perpendicular to the fibril orientation. The solid lines represent the spectra obtained within tactoids, while the dotted lines represent the spectra obtained outside of tactoids. See Figure S1, Supporting Information for the corresponding fluorescence images.

Rich liquid crystal phases are formed from the self‐assembly of amyloid fibrils suspended in water. At 2 wt%, tactoids where orientationally ordered fibrils concentrate, emerge spontaneously from the isotropic surrounding. The process is driven by entropy facilitated by the large aspect ratio of the fibrils (averaging ≈600 nm long and ≈4 nm wide, see the Experimental Section for the sample preparation) and leads to both nematic and cholesteric configurations in the tactoids.^[^ ^15^, ^16^ ^]^ The interplay between bulk elastic energy and the surface conditions give rise to a variety of shapes and structures of tactoids, including homogeneous, bipolar, cholesteric, etc., each showing unique texture under a polarizing optical microscope (POM) (**Figure** **1**). The patterns are results of the birefringence originating from the collective orientation of the fibrils and can be used to deduce the local fibrils orientation, namely the director field **n**(**r**).^[^ ^15^, ^16^ ^]^ Similar patterns with varying intensity corresponding to the director distributions are also observed by polarized fluorescence microscopy (Figure 1a–d). Combined with the director distribution of the imaged tactoids, the fluorescence microscopy images reveal the polarization dependence of the AFs’ intrinsic fluorescence: under the excitation of a linearly polarized laser of 405 nm, the microscopy images show maximized fluorescence in the region where the polarization is parallel to the orientation of the fibrils and minimized fluorescence where they are orthogonal to each other. Rotating the tactoids with respect to the polarization of the excitation laser thus changes the contrast and intensity distribution, as shown in Figure 1b,d. The white stripes that correspond to the cholesteric fingerprint texture can disappear entirely when they are placed perpendicular to the polarization direction. The spectra measured at different polarizations capture the behavior in more detail. To minimize the effect of varying alignment of the fibrils, the spectra are collected from either a homogeneous tactoid or a small region at the center of a large bipolar tactoid (Figure S1, Supporting Information). As shown in Figure 1, the aligned regions show stronger fluorescence compared to that of the isotropic regions (colored solid lines vs colored dotted lines) when the polarization is parallel to the fibrils alignment, and the aligned regions show stronger fluorescence when the polarization is parallel to the fibrils orientation compared to when the two are orthogonal (colored solid lines vs black dotted lines). It is worth noting that the stronger fluorescence intensity of the tactoid may partly come from the higher concentration of the fibrils in the aligned region, i.e., within the tactoids, compared to that outside in the isotropic surrounding.^[^ ^12^, ^16^ ^]^ However, the reduced fluorescence intensity when the polarization is perpendicular to the fibril orientation clearly demonstrates the polarization sensitivity of the AFs’ intrinsic fluorescence. In addition to the 405 nm excited blue‐green luminescence that is characteristic of amyloids,^[^ ^17^, ^18^, ^19^, ^20^, ^21^, ^22^ ^]^ this polarization dependence is also observed in the fluorescence under the excitation of longer wavelengths, such as 514 and 633 nm (Figure 1f,g). As previously reported, amyloid fibrils fluoresce when excited by a wide range of wavelength of lights.^[^ ^18^ ^]^ Our result here shows that the polarization dependences is also not limited to the blue‐green luminescence, but may extend to a similarly wide range of the spectrum.

**Figure 2:**
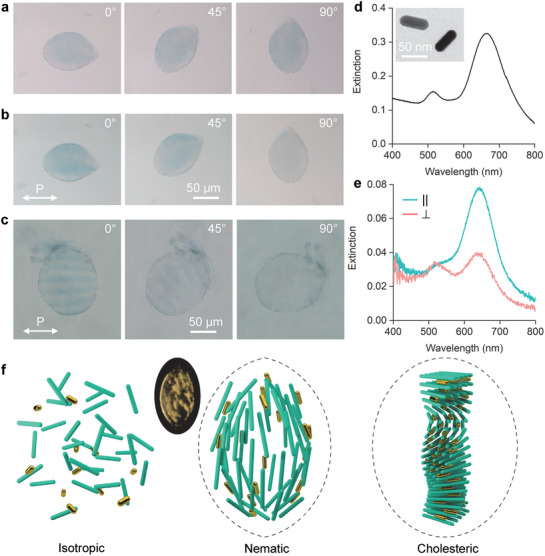
Amyloid fibril (AF)‐gold nanorod (GNR) hybrid tactoids. a) Microscopy images showing the color of a hybrid tactoid rotated under unpolarized illumination light. b) The same tactoid with a fixed polarization of illumination light (marked with P). c) Microscopy images of a cholesteric hybrid tactoid under linearly polarized light. d) Extinction spectrum of PEG‐capped GNRs dispersed in water. The inset shows an SEM image of the GNRs used. e) Polarization‐dependent extinction spectra of a hybrid tactoid. See the corresponding microscopy images in Figure S5 (Supporting Information). f) Schematics of AF‐GNR codispersion in isotropic, nematic, and cholesteric phases. The latter two are shown in bipolar and cholesteric tactoids. The long green rods represent the AFs and the short golden rods represent the GNRs; the schematics are not true to scale. The inset is a 3D reconstruction of a bipolar hybrid tactoid from a stack of fluorescence images as in Figure S4, Supporting Information.

To explore the possibility of enhancing the fluorescence signal of the AFs, we introduce plasmonic nanoparticles into the system. After surface functionalization with a polymer, thiol‐terminated methoxy‐poly(ethylene glycol) (mPEG‐SH),^[^ ^23^, ^24^ ^]^ the GNRs are added to the aqueous fibrils suspension, mixed well and left undisturbed. Self‐assembly again drives the formation of tactoids, but unlike the case of fibrils only, some of the tactoids have a distinctive blue tint, indicating a high concentration of GNRs within (**Figure** **2**; and Figure S2, Supporting Information). The phase behavior of these hybrid tactoids is otherwise very similar to that of pure fibrils tactoids,^[^ ^15^, ^16^ ^]^ exhibiting a variety of shapes and director field configurations as seen in suspensions of only fibrils, including cholesteric tactoids. The overall concentration of the fibrils remains about 2 wt%, with the addition of the GNR suspension and the loss of water in the process taken into account. Under crossed polarizers, the same birefringent patterns are also observed, in addition to some red/golden lines and dots around the hybrid tactoids, which are GNR aggregates at the interfaces and can also be captured with a fluorescence microscope (Figures S2–S4 and Video S1, Supporting Information).

An interesting discovery is that the GNRs within the tactoids are aligned, and their alignment follows the local director field of the fibrils. The collective orientation of GNRs is revealed by the color change of the hybrid tactoids when the linear polarization of the illumination light is varied (Figure 2b), which corresponds to the switching of the dominant absorption peaks in the extinction spectra (Figure 2e; and Figure S5, Supporting Information). The behavior can be understood as the selective activation of the SPR absorption peaks of the GNRs enabled by their orientational ordering in the hybrid tactoids.^[^ ^23^, ^24^ ^]^ When the polarization of the incident white light is parallel to the long axis of the GNRs (the direction of which is defined by the local director orientation), the longitudinal SPR absorption band around 660 nm is dominant, resulting in strong absorption in the red range of the spectrum and the tactoid appears blue in the transmission mode of the optical microscope. In contrast, when the polarization of the light is orthogonal to the long axis of the GNRs, the weaker transverse peak is present but not strong enough to cause a dramatic color tint as in the previous scenario. Thus, when compared with the POM images of the tactoids, the orientations of the GNRs inferred from the color change are seen to follow the director orientation of the hosting LC, and are in fact imposed by the aligned fibrils through steric interactions.^[^ ^23^, ^24^ ^]^ The GNRs can follow more complex director configurations such as that in a cholesteric tactoid–blue stripes are observed across the tactoid and they disappear when rotated 90° (Figure 2c). The stripes are essentially the fingerprint texture in a typical cholesteric tactoid as also observed in the birefringent patterns under the POM (Figure S3, Supporting Information). The cholesteric pitch measured in both cases is about 38 µm, comparable to that measured in the tactoid of only fibrils. The observations indicate that the GNRs are indeed following the helical twisting structure of director field in a cholesteric hybrid tactoid (see Figure 2f for a schematic illustration).

**Figure 3:**
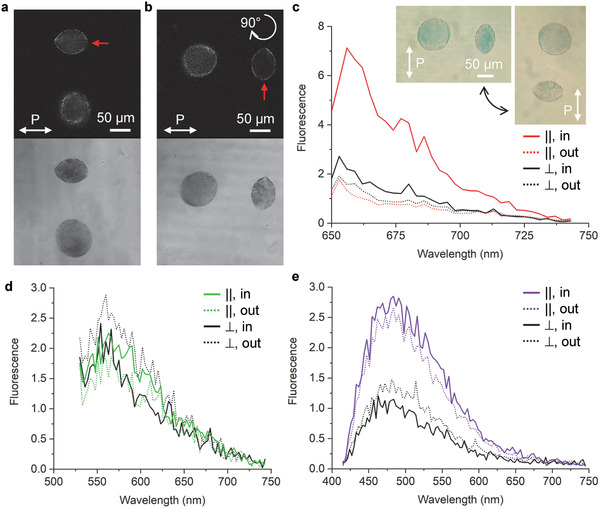
Plasmonic enhanced fluorescence of AF‐GNR hybrid tactoids. a) Fluorescence image of hybrid tactoids when the polarization of the excitation light (633 nm) is parallel to the long axis of a bipolar tactoid. Inset at the bottom shows the image obtained in transmission mode. b) Fluorescence and transmission microscopy images of the tactoids in (a) rotated 90°. c–e) Polarization dependent fluorescence spectra measured within and outside of a hybrid tactoid (marked with the red arrows in (a) and (b) using 633 nm (c), 514 nm (d), and 405 nm (e) excitation light. The lines are color‐coded and dashed the same way as in Figure 1.

The hybrid tactoids exhibit enhanced fluorescence (**Figure** **3**; and Figure S6, Supporting Information). Excited by a linearly polarized 633 nm laser, the hybrid tactoids stand out against the isotropic background with bright interior and clear boundaries in the fluorescence channel (Figure 3a,b). In the transmission channel, the tactoids cast a much darker shadow because of the strong absorption and scattering from the GNRs. Meanwhile in the same microscopy image, the other tactoids with little amount of GNRs are almost invisible compared to the hybrid ones. This enhancement is also shown on the fluorescence spectra (Figure 3c). By integrating the area under the spectra curves, we find that the fluorescence signal within the tactoid is 4.4 times of that measured outside in the isotropic region (corresponding to the solid and dotted red lines in Figure 3c, respectively). In the system of only AFs and no GNRs, the same ratio is only 1.2 (obtained from Figure 1g). We also observe that the enhancement depends strongly on the polarization and wavelength of the excitation light. When the hybrid tactoid is rotated 90°, the fluorescence signal from within the tactoid is reduced to be comparable to the one from the isotropic surrounding. At other wavelengths such as 514 and 405 nm used here, the enhancement is much less pronounced, if present at all (Figure 3d,e, compare the colored solid and dotted lines).

The fluorescence enhancement with its dependence on the polarization and wavelength of light can be understood from the plasmonic effect of GNRs coupled with the intrinsic luminescence of the amyloid fibrils. Briefly, the GNRs amplify the local electromagnetic field due to the SPRs when the frequency of the incident light matches with the SPR oscillating frequency.^[^ ^25^, ^26^ ^]^ Therefore, the amyloid fibrils in the vicinity of GNRs experience a locally heightened excitation field and emit stronger fluorescence signals. Although considered very weak, the SPR‐induced luminescence from the GNRs may also contribute to the fluorescence signal.^[^ ^27^, ^28^ ^]^ The longitudinal and transverse SPR modes of the GNR used in this study corresponds to incident wavelengths of ≈660 and 514 nm, and are activated by the light with polarization parallel and perpendicular to the long axis of the GNR, respectively. The enhancement from the two SPR modes, however, is vastly different, with the field intensity produced by the longitudinal mode much stronger than the transverse mode.^[^ ^25^, ^26^, ^27^, ^28^ ^]^ This theoretical analysis qualitatively agrees with our experimental observations (compare Figure 3c,d).

**Figure 4:**
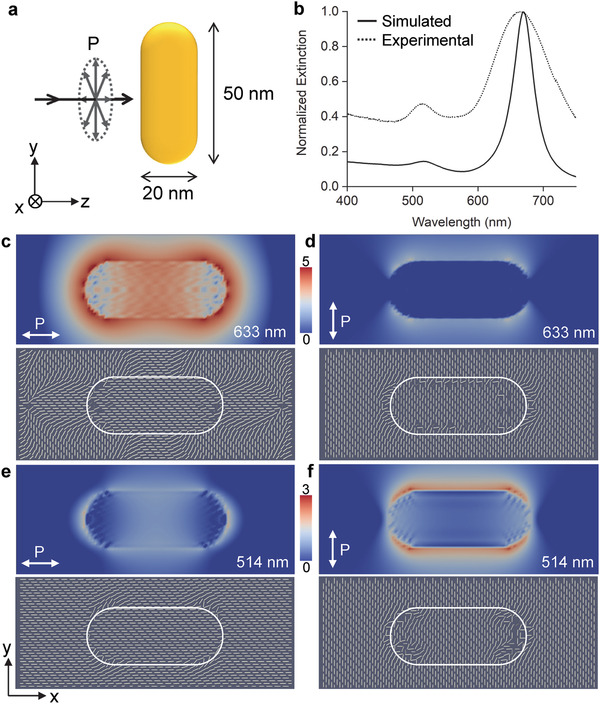
Numerical simulations of plasmonic responses of GNRs. a) Schematic showing the parameters and setup for the simulations. The dimensions of the GNRs are 20 × 50 nm and plane‐wave incident light with varying polarizations is used as the excitation. b) Simulated and experimental extinction spectra of GNRs dispersed in water. c–f) Simulated near‐field enhancement of a GNR excited with 633 and 514 nm light. The colors represent the order of magnitude of electric field enhancement, i.e., log(*E* ^2^/*E*_0_^2^); note the different maximum values when excited with 633 and 514 nm. The incident light propagates along *z* axis as illustrated in (a) and the ensuing field distribution is extracted as the central cross‐sections in the *xy* plane. The insets at the bottom of each panel are the simulated distribution of polarization with the white dashes representing the local direction of polarization; the capsule shapes in the insets indicate the boundary of the GNR. The size of the simulated areas is 40 × 100 nm^2^; the incident polarization *P*, defined along the direction of the electric field, is marked with white double arrows.

To better understand the interaction between the plasmonic enhancement of the GNRs and the fibrils, numerical simulations are performed to find the details of the field distribution around GNRs under different excitation conditions. By first matching with the experimental measurement of the size and extinction spectrum of GNRs, the field intensity and polarization distributions in the close vicinity of a GNR are obtained at varying wavelengths and polarizations (**Figure** **4**). The simulation shows that the enhancement is a near‐field effect, consistent with previous studies.^[^ ^25^, ^26^ ^]^ As shown in the simulated intensity distribution with 633 nm excitation, strong enhancement (defined as *E* ^2^/*E*_0_^2^ > 10^2^) happens mostly within a shell of less than 10 nm away from the surface of the GNR. Therefore, it is important that the distance between the fibrils in LC phases is small enough such that they can still experience the enhanced field from the GNRs suspended in between (see the schematic of Figure 2f). The inter‐fibrils distance can be estimated using the Onsager critical concentration^[^ ^12^ ^]^ ϕ ≈ 4*D*/*L*, where ϕ is the volume ratio of the fibrils in the aqueous suspension, *L* = 600 nm and *D* = 4 nm are the length and the diameter of the fibrils. Assuming a square lattice distribution of the fibrils, the volume ratio can also be expressed as ϕ ≈ *πD* ^2^/4*d* ^2^, where *d* is the core‐to‐core distance between the fibrils and is computed to be 22 nm. Given that the diameter of the GNRs is around 20 nm, the distance between the GNRs and the fibrils is thus sufficiently small for the enhancement. In comparison, at 514 nm and when the polarization is perpendicular to the GNR axis, the region with strong enhancement is confined to less than 3 nm away from the GNR surface, allowing for much less overlap with the fibrils. Additionally, the fibrils will be perpendicular to the excitation polarization. Therefore, in experimental measurements, these factors combined lead to less pronounced fluorescence at 514 nm and also at other wavelengths away from either of the SPR modes such as 405 nm (Figure S7, Supporting Information). On the other hand, should an excitation laser closer to the GNR's longitudinal SPR wavelength be used, we may expect even stronger fluorescence enhancement compared to that at 633 nm. These SPR‐determined effects can be tuned by adjusting the aspect ratio of the GNRs or the shape and composition of the plasmonic nanoparticles to match with desired excitation wavelengths,^[^ ^25^, ^26^ ^]^ which may facilitate imaging amyloid aggregates in situ in the near‐IR range.

Despite the similarities between our system and other hybrid liquid crystal colloids such as cellulose nanocrystals (CNCs) and GNRs,^[^ ^23^, ^29^ ^]^ the key difference observed here is the tactoids with concentrated doping nanoparticles. In the previously reported CNC‐GNR codispersions, GNRs tend to be more concentrated in the isotropic region of the biphasic colloidal suspension.^[^ ^23^, ^29^ ^]^ Theoretical studies on binary mixtures of rod‐like particles with different lengths also point out that longer rods tend to have higher concentrations in the anisotropic phase, while shorter ones have higher concentrations in the isotropic phase.^[^ ^30^, ^31^ ^]^ Therefore, it is unexpected and unconventional that in this system the GNRs, being much shorter than the fibrils, have a higher number density within the tactoids compared to that outside in the isotropic phase. This can be inferred from the blue‐tinted and fluorescent hybrid tactoids that have a sharp contrast to the surrounding isotropic region. However, it is worth noting that most of the theoretical treatments of the binary mixture of rods are based on the Onsager theory assuming large aspect ratios of the rods and rigid interparticle interactions. These assumptions are not necessarily true in this system given the small aspect ratios of the GNRs used and that the proteinaceous fibrils may interact with the GNRs in complex ways. Because amyloid fibrils are absorptive to a wide range of nanoparticles,^[^ ^7^, ^8^, ^9^, ^10^ ^]^ it is possible that the attractive force between GNRs and the fibrils lead to the congregation of GNRs and fibrils within the hybrid tactoids. The apparent discrepancy between this result and widely known theories calls for further investigation to uncover the physical underpinnings.

Fluorescence anisotropy is common among fluorescent materials, given that polarized light selectively excites the transition moments that are (roughly) parallel to the polarization and that the excited fluorophores then emit polarized signals.^[^ ^32^ ^]^ Here, with the help of the LC orientational ordering, the orientation of the transition moments within the fibrils are mostly preserved instead of being completely randomized by the Brownian motion. Therefore, rotating the polarization with respect to the alignment of the fibrils is equivalent to rotating with respect to the orientation of the fluorescence transition moments. The contrast of the fluorescence strength at different excitation polarizations suggests that the transition moments largely orient parallel to the fibril backbones, and less along the β‐strands that run perpendicular to the fibrils. This finding may help to find the origin of the luminescence of amyloid fibrils across a wide range of the spectrum. Some early studies have related the blue‐green luminescence to the aromatic residues in the fibrils, but more recent ones infer that it is a structural property related to the extensive hydrogen bond networks that connects the cross‐β‐structure.^[^ ^17^, ^18^, ^19^, ^20^, ^21^, ^22^, ^33^, ^34^, ^35^ ^]^ Our results support this hypothesis as the hydrogen bonds and the related intramolecular structures are largely parallel to the fibril axis, being orthogonal to the β‐strands. We expect that exploring the fluorescence anisotropy of amyloid fibrils from other protein sources and in various environments can provide a definite answer to this question. More in general, exploiting the liquid crystalline orientation ordering may be instrumental to uncover the fluorescence anisotropy mechanisms in other proteinaceous materials.^[^ ^36^ ^]^

## 3 Conclusion

We have demonstrated hybrid tactoids consisting of GNRs aligned by amyloid fibrils in LC mesophases. The plasmonic inclusions with orientational ordering lead to the selective absorption of light and enhanced fluorescence with polarization sensitivity. While this system shares similarity to previous reported LC guest–host system, such as CNC‐GNRs, the fundamental difference in this work is that the concentration of GNRs is higher in the tactoids compared to the isotropic region, suggesting a different kinetic process of the self‐assembly and calling for deeper analysis of its physical underpinnings. This organic–inorganic hybrid codispersion from the self‐assembly of amyloid fibrils and GNRs may be extended to anisotropic colloidal particles of other shapes and composition with desirable optical, magnetic, and electronic properties.^[^ ^37^ ^]^ The colloidal inclusions can also be synthesized directly within the amyloid suspension^[^ ^38^ ^]^ and employing AFs made from plant‐based proteins can help producing environmentally friendly functional materials with pre‐engineered properties.

## 4 Experimental Section

### Synthesis of Amyloid Fibrils

AFs were synthesized using β‐lactoglobulin sourced from whey protein of bovine milk following an established protocol.^[^ ^15^, ^16^ ^]^ First, 6 g of purified β‐lactoglobulin was dissolved in 294 mL of Milli‐Q water to make a 2 wt% solution and the pH was adjusted to 2. The solution was then incubated at 90 °C for 5 h and quenched in ice‐water mixture to stop the reaction, followed by the application of mechanical shearing to shorten the fibrils. The resulting solution was further purified by dialysis in 10 L of pH 2 Milli‐Q water for 5 days (water changed daily) and then upconcentrated to 2 wt% by reverse osmosis in 1 L of 6 wt % PEG (Sigma‐Aldrich, MW 35 kDa) solution adjusted to pH = 2. The tubing used in the purification and upconcentration was Spectra/Por dialysis membrane 1 with molecular‐weight cutoffs of 100 and 6–8 kDa, respectively. This process yielded AFs averaging 600 nm long and 4 nm wide.^[^ ^16^ ^]^

### Synthesis and Grafting of Gold Nanorods

GNRs are synthesized using a seed‐mediated method.^[^ ^39^ ^]^ To prepare the seed solution, 0.1 mL of 0.025 m HAuCl_4_ solution was mixed with 10 mL of 0.1 m aqueous cetyltrimethylammonium bromide (CTAB), followed by injecting 0.6 mL of 0.01 m ice‐cold NaBH_4_ solution with vigorous stirring for 2 min. The seed solution was aged for 30 min before using. The growth solution was prepared by combining 0.2 mL of 0.025 m HAuCl_4_, and 0.04 mL of 0.016 m AgNO_3_ solutions with 10 mL of 0.1 m CTAB solution with gentle mixing. The subsequent addition of 0.09 mL of 0.08 m ascorbic acid solution turned the mixture from yellow to colorless. After 20 min, 12 µL of the seed solution was added and the ensuing solution was kept undisturbed at 30 °C for 12 h. The final dispersion containing GNRs showed dark turquoise color. To functionalize the surface of GNRs with mPEG‐SH (Jenkem, MW 5 kDa), the dispersion was centrifugated at 10 000 rpm for 10 min and redispersed in 2 mL of 30 mg mL^−1^ PEG in Milli‐Q water. This process was repeated twice with 24 h intervals to ensure successful grafting of PEG on the GNRs. The extinction spectrum of the GNR dispersion was collected with a UV–vis spectrophotometer (Cary 100 Bio). Unless noted otherwise, all the chemicals were obtained from Sigma‐Aldrich and used without further purification.

### Preparation of AF‐GNR Codispersions

100 µL of the PEG‐capped GNR dispersion was washed in pH = 2 Milli‐Q water three times by centrifugation at 9000 rpms for 10 min to remove the excess PEG and match with the pH of the AF suspension. After removing the supernatant from the last centrifugation, 5 µL of the remaining GNR dispersion was combined with 35 µL of the AF solution with gentle mixing by a pipet. The mixture was then infiltrated in a glass capillary tube with rectangular cross‐section measuring 4 × 0.2 mm^2^ and 5 cm in length. One end of the capillary tube was sealed with UV‐curable glue, while the other end was left open. The sample was left undisturbed for 2–3 days, during which the fibrils and nanorods self‐assemble into the hybrid tactoids. Samples containing only AF dispersions were prepared in the same type of glass capillary tubes without adding GNRs.

### Sample Characterization

The polarizing optical microscopy images of the AF tactoids with and without GNRs were captured using an upright microscope (Zeiss AxioImager.Z2) with 5× or 20× objective lens. The polarization dependence fluorescence images and spectra were collected on a confocal microscope (Leica TCS SP8) with a 20× objective (0.75NA HC PLAN APO CS2). The excitation source used in the fluorescence microscopy and spectroscopy were continuous laser lines of 405, 514, 458, and 633 nm with the power in the range of 0.2–4 mW. The laser lines were chosen to best excite the intrinsic luminescence of the AFs as well as to match with the SPR frequencies of the GNRs. The polarization‐dependent extinction spectra of the hybrid tactoids were measured by confining the polarized illumination with a pinhole using a homebuilt setup,^[^ ^40^ ^]^ enabling the confocal illumination of single tactoids. The reference of the extinction measurement was collected from the isotropic surroundings, which contains less concentrated GNRs as indicated by the spectra.

### Numerical Simulation

The discrete dipole approximation method was used to simulate the field intensity and polarization distribution in the vicinity of a GNR upon excitation of electromagnetic waves of different wavelengths.^[^ ^41^, ^42^, ^43^, ^44^ ^]^ The free computational resources including an open source tool (nanoDDSCAT) were accessed on the website nanohub.org.^[^ ^45^ ^]^ In the simulations, the GNR was modeled with the built‐in shape profile of a cylinder with end‐caps. The length of the cylinder was set to be 30 nm and the diameter of the hemispherical caps was 20 nm with a dipole density of 1 dipole per nm. The GNR was rotated with respect to the direction of the incoming light to simulate it being excited by light of different polarizations (Figure 4a). The refractive index of water 1.33 was used as that of the surrounding medium given that the system was aqueous suspensions. First the extinction spectra of GNRs in the visible range (400–800 nm) were simulated to confirm that the simulated spectra agree with the experimental measurement of GNR suspension in water. Then four wavelengths, 405, 514, 633, 670 nm were chosen for simulating the field distribution due to the SPR effect of the GNR. They correspond to the excitation lasers used in this study as well as the SPR frequencies of the GNR simulated. Note that the longitudinal SPR wavelength of the simulated GNR is 670 nm, slightly different from that of the GNRs used in the experiments (660 nm), but the small difference does not affect the comparison between the simulation and experiments.

## Conflict of Interest

The authors declare no conflict of interest.

## Supporting information

Supporting Information

Supplemental Video 1

## Data Availability

The data that support the findings of this study are available from the corresponding author upon reasonable request.
