# Supplementary material for: Plasmonic Amyloid Tactoids
Source: Adv Mater. 2021 Oct 17;33(51):2106155. doi: 10.1002/adma.202106155 (PMC11468577; doi:10.1002/adma.202106155)
Supplement: Supplementary file 1 — Supporting Information [file ADMA-33-2106155-s002.pdf]

ADVANCED MATERIALS

Supporting Information

for *Adv. Mater.,* DOI: 10.1002/adma.202106155

Plasmonic Amyloid Tactoids

*Ye Yuan, Hamed Almohammadi, Julie Probst, and Raffaele Mezzenga**

Supporting Information

Plasmonic Amyloid Tactoids

*Ye Yuan, Hamed Almohammadi, Julie Probst and Raffaele Mezzenga**

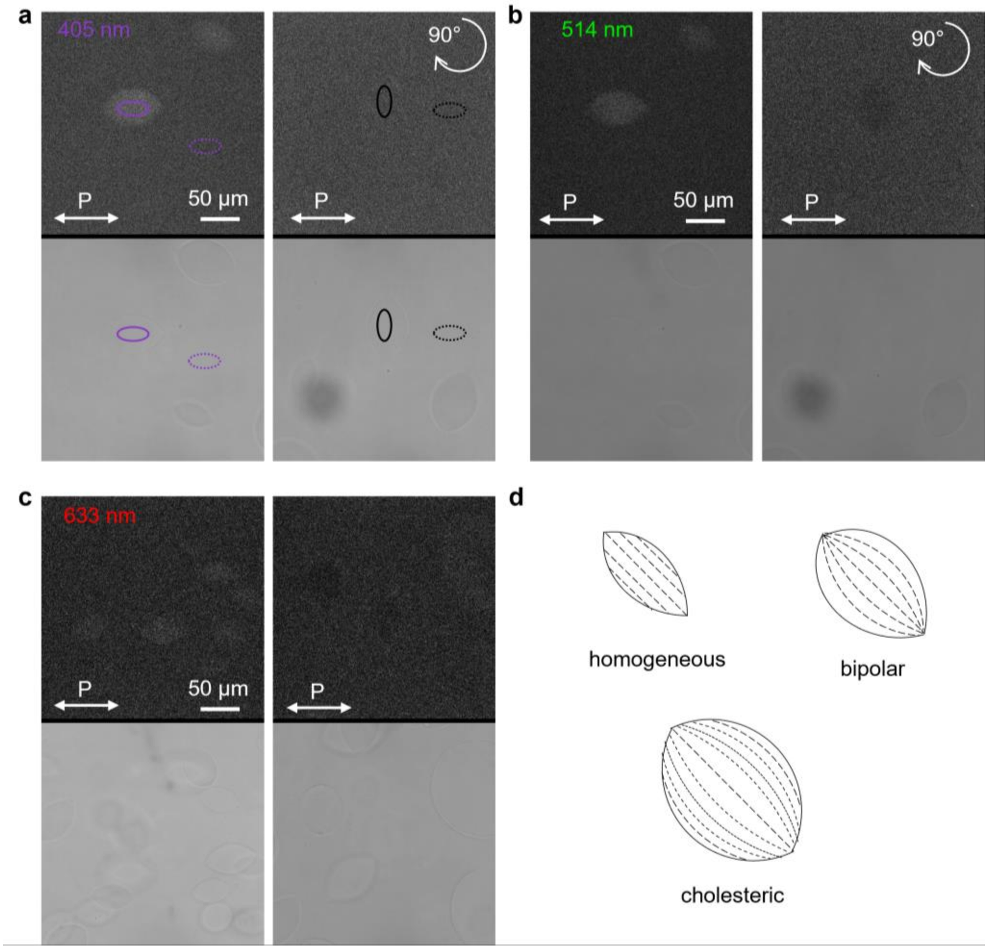

Figure S1. Fluorescence images with polarization dependence corresponding to the spectra in Figure 1. As an example shown in (a), the circles show where the spectra from within and outside of a tactoid are collected. The sample is then rotated 90° to collect spectra from within and outside of the same tactoid again. The spectra are collected the same way in (b), while in (c) spectra are collected from two tactoids with their long axis parallel (left) and perpendicular (right) to the incident polarization. The accompanying images obtained in transmission mode (insets at the bottom of each panel) are used to cross-check the location and boundary of the tactoids of interest. (d) Schematics showing the director orientation within different types of tactoids.

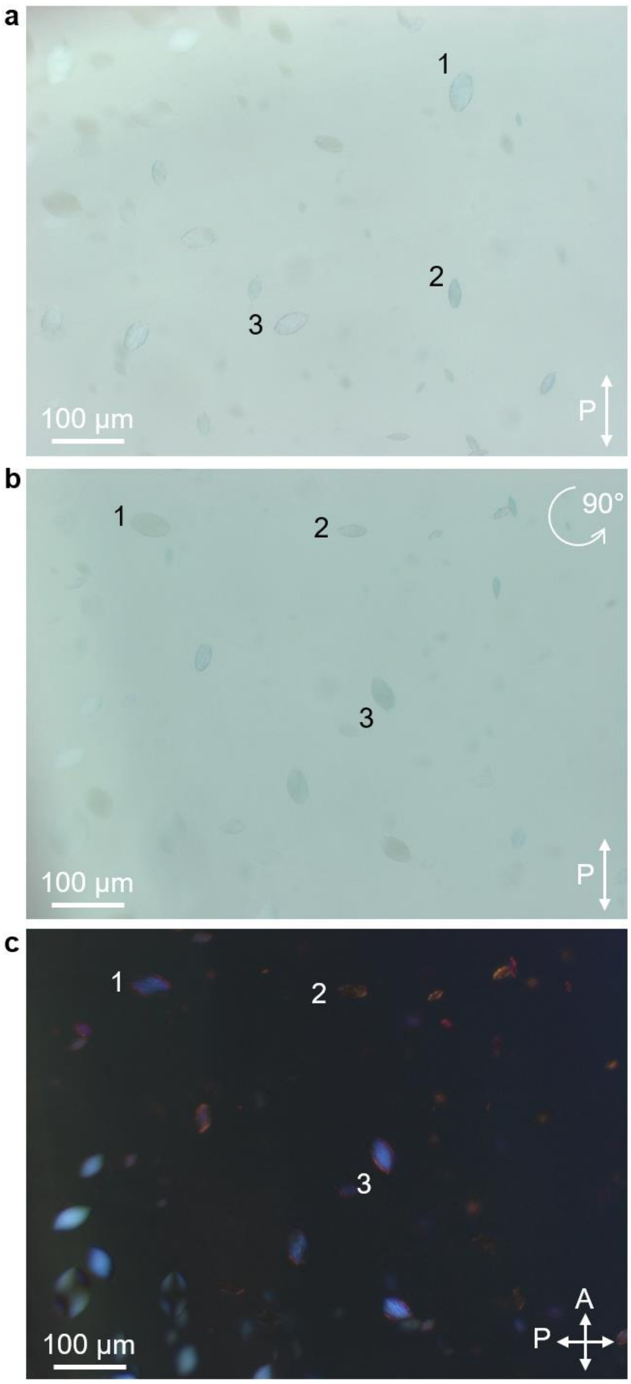

Figure S2. Polarizing optical micrographs of AF-GNR hybrid tactoids and regular AF tactoids. The sample is placed under linearly polarized light (a), rotated 90° (b), and then observed under crossed polarizers (c). The white double arrows indicate the direction of the polarizer P and analyzer A. Three hybrid tactoids are marked as the references in the images. Compared to regular tactoids, the hybrid ones are characterized by their blue tint under linearly polarized light and red-golden boundaries under crossed polarizers.

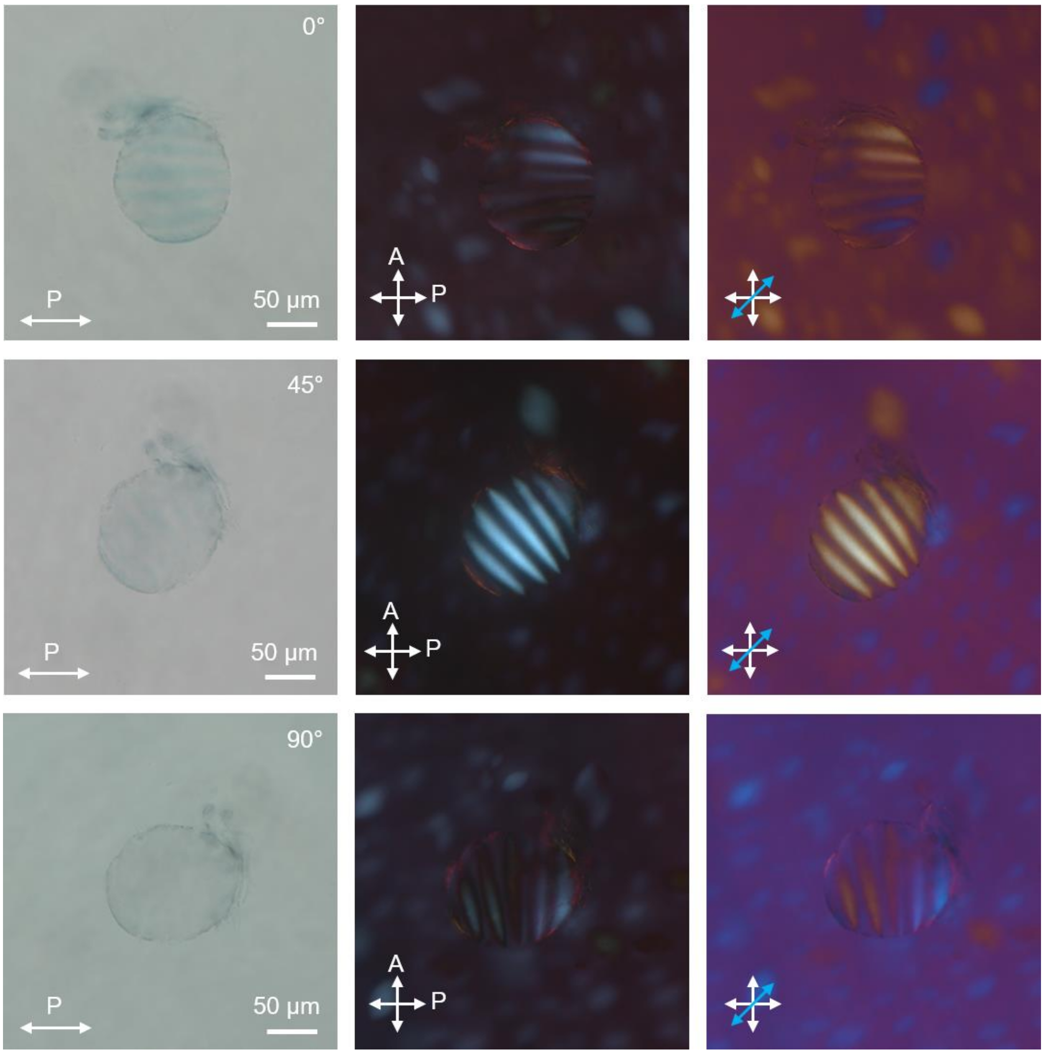

Figure S3. Polarizing optical micrographs of a AF-GNR hybrid tactoid in cholesteric phase. From left to right, the tactoid is characterized under linearly polarized light, crossed polarizers, and crossed polarizers with a 530 nm retardation plate. From top to bottom, the sample is rotated clockwise 90°. The white double arrows indicate the direction of the polarizer P and analyzer A; the slow axis of the retardation plate is marked with the blue double arrow. Note that the colors in the micrographs of the right column result from the insertion of the retardation plate and are related to the LC director orientations.

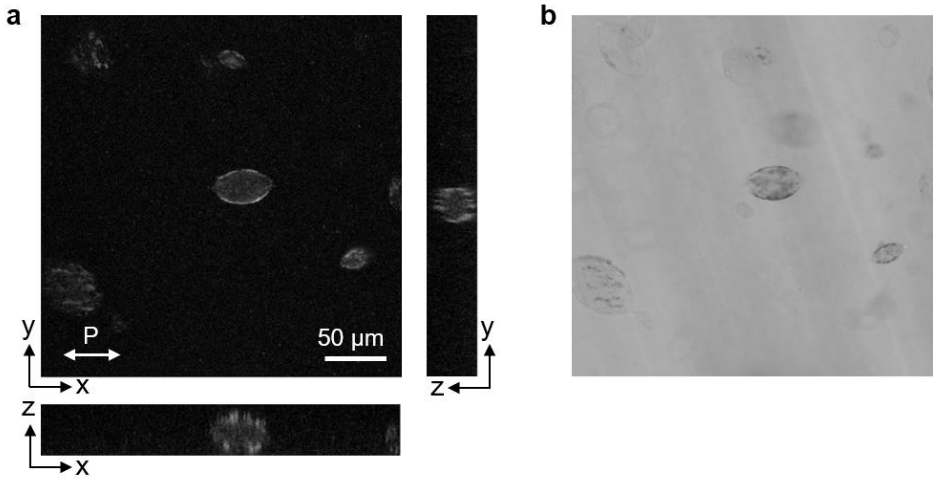

Figure S4. Slices from a 3D fluorescence image of hybrid tactoids. (a) Images obtained by cross-sectioning the central hybrid tactoid on the *xy*, *yz*, and *xz* plane. The white double arrow indicates the polarization direction of the 633 nm excitation light. (b) Corresponding transmission images in *xy* plane. See Video S1, Supporting Information for a video going through all the fluorescence micrographs of the 3D image.

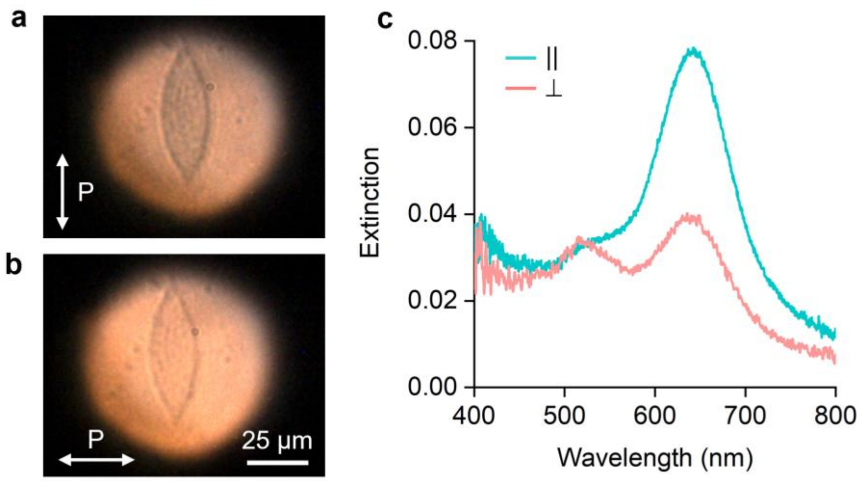

Figure S5. Extinction spectra measurement of an AF-GNR hybrid tactoid. (a,b) Micrographs of the tactoid locally illuminated with polarized light. The polarization of the illuminating light P is marked with the white double arrows. (c), corresponding spectra as in Figure 2e. The spectrum measured when the polarization is parallel to the long axis of the tactoid corresponds to (a), and the spectrum measured when the polarization is perpendicular to the long axis of the tactoid correspond to (b).

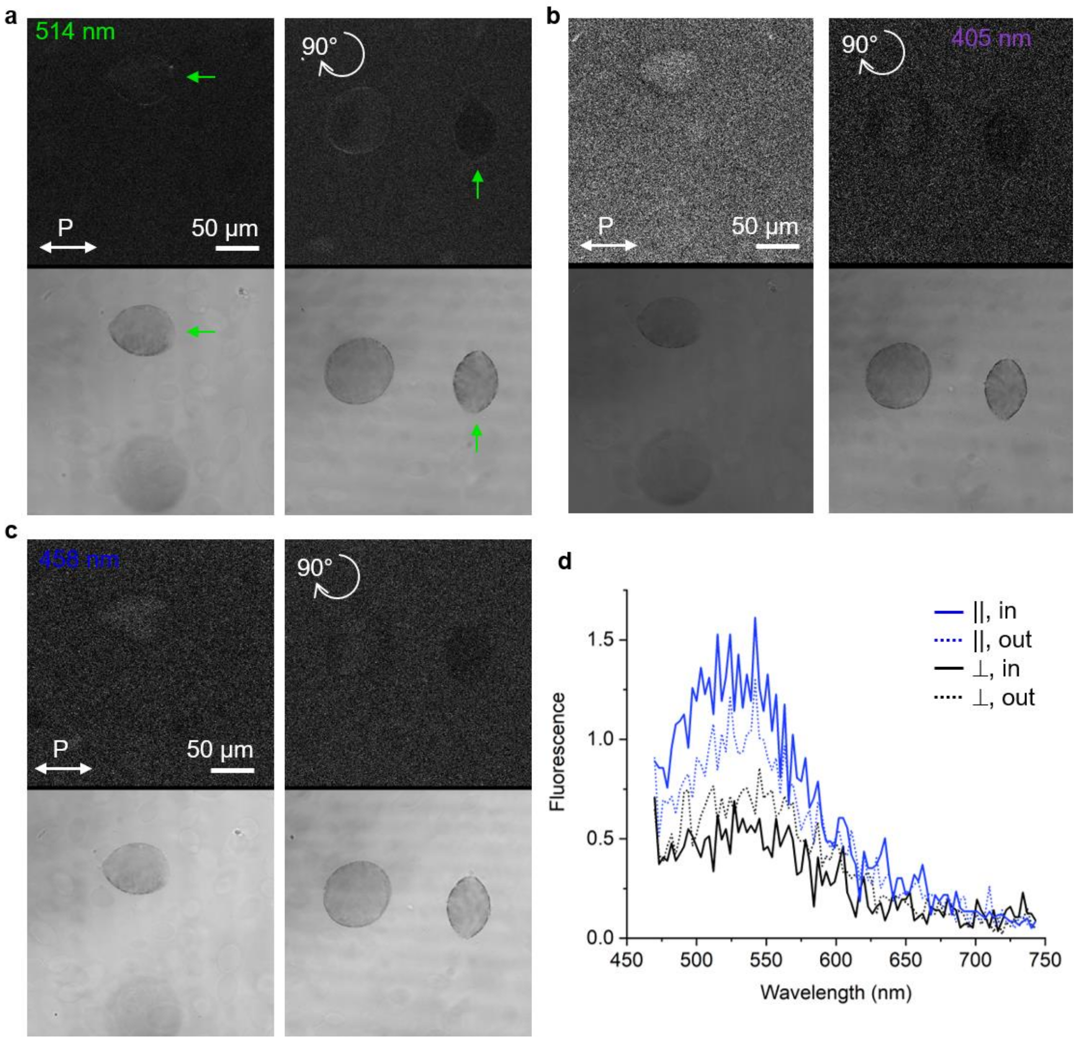

Figure S6. Polarization dependent fluorescence of hybrid tactoids. (a-c) Fluorescence images obtained with 514 nm (a), 405 nm (b), and 458 nm (c) excitation light; the polarization of the excitation light is marked with the white double arrow. (a) and (b) are the corresponding images to the spectra in Figure 3d and 3e. (d) Polarization dependent fluorescence spectra corresponding to (c). The tactoid of interest is marked with the green arrows in (a) (the same one as in Figure 3), and the spectra are collected in the same method as described in Figure S1.

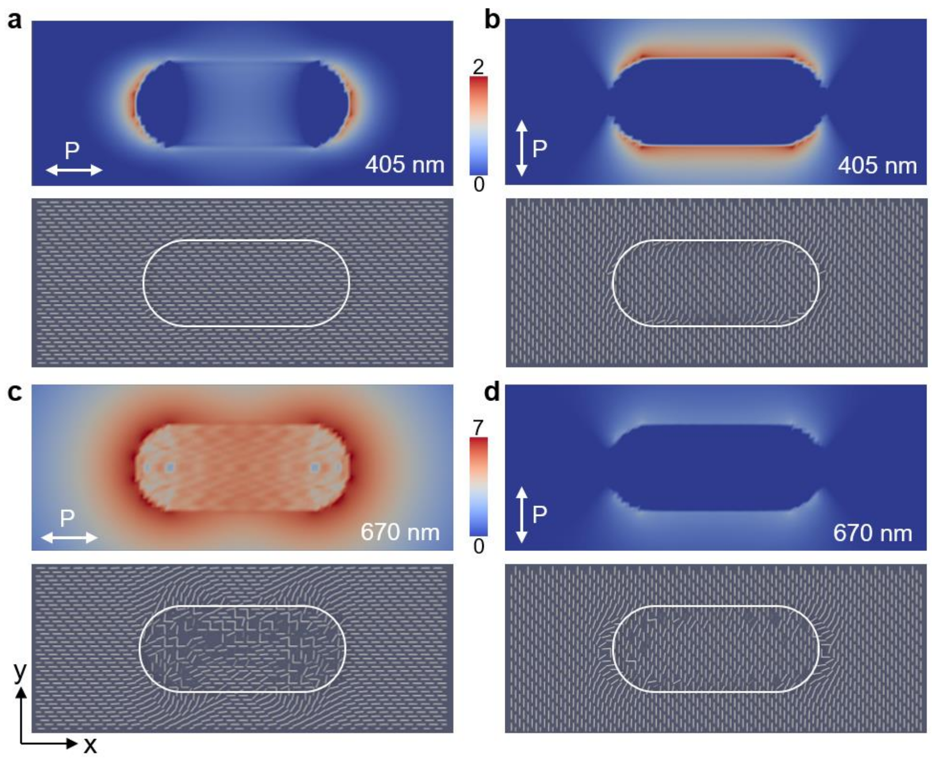

Figure S7. Simulated near-field enhancement of a GNR excited with 405 nm and 670 nm light. The same model as in Figure 4 is used except for the excitation wavelength. Colors represent the order of magnitude of the electric field enhancement, i.e. log(E^2/E_0^2). The incident polarization P, defined along the direction of the electric field, is marked with white double arrows. Insets at the bottom of each panel are the simulated distribution of polarization with the white dashes representing the local direction of polarization.

Video S1. 3D fluorescence image of hybrid tactoids. The video shows in sequence the fluorescence micrographs obtained by advancing the focal plane in the direction perpendicular to the field of view, allowing for reconstructing the tactoids in 3D. See vertical cross sections in Figure S4, Supporting Information. The excitation wavelength is 633 nm with the polarization direction marked by the white double arrow.
